# Software project management tools in global software development: a systematic mapping study

**DOI:** 10.1186/s40064-016-3670-7

**Published:** 2016-11-24

**Authors:** Saad Yasser Chadli, Ali Idri, Joaquín Nicolás Ros, José Luis Fernández-Alemán, Juan M. Carrillo de Gea, Ambrosio Toval

**Affiliations:** 1 Software Project Management Research Team, ENSIAS, Mohammed V University, Rabat, Morocco; 2 Department of Informatics and Systems, Faculty of Computer Science, University of Murcia, Murcia, Spain

**Keywords:** Software project management, Global software development, SPM tool, Systematic mapping study

## Abstract

Global software development (GSD) which is a growing trend in the software industry is characterized by a highly distributed environment. Performing software project management (SPM) in such conditions implies the need to overcome new limitations resulting from cultural, temporal and geographic separation. The aim of this research is to discover and classify the various tools mentioned in literature that provide GSD project managers with support and to identify in what way they support group interaction. A systematic mapping study has been performed by means of automatic searches in five sources. We have then synthesized the data extracted and presented the results of this study. A total of 102 tools were identified as being used in SPM activities in GSD. We have classified these tools, according to the software life cycle process on which they focus and how they support the 3C collaboration model (communication, coordination and cooperation). The majority of the tools found are standalone tools (77%). A small number of platforms (8%) also offer a set of interacting tools that cover the software development lifecycle. Results also indicate that SPM areas in GSD are not adequately supported by corresponding tools and deserve more attention from tool builders.

## Background

Globalization as an economic and social trend has relentlessly pushed businesses to turn from national markets toward a global market in which new forms of concurrence and cooperation have been spawned. In the last decade, software development in particular has undergone a drastic change in its business operations and processes. This concerns not only marketing and distribution but also the way in which software is conceived, designed, constructed, tested, and delivered to customers (Herbsleb and Moitra 2001). In particular, global software development (GSD) is now an expanding trend in the software industry (Santos et al. 2012) owing to the advantages that it may have over collocated software development (Conchúir et al. 2006, 2009). It helps software companies access highly qualified resources at a lower cost, provides them with easier access to customers and allows 24/7 work shifts. However, it also adds new challenges to the management of the already complex software projects (da Silva et al. 2010). The geographic separation of teams, the social and cultural differences among people, along with time zone differences, greatly impact on communication and collaboration and limit the success of projects in a highly distributed environment (Steinmacher et al. 2013; Portillo-Rodríguez et al. 2012).

Research on GSD has increased since it is becoming an effective technique (Haq et al. 2011) and existing descriptions of the software project management (SPM) field (PMI 2004; Abran et al. 2001) do not consider globalization explicitly. This has therefore led to a new need to study and assess the downsides of GSD in SPM and to provide new methods, techniques and tools with which to alleviate them. As a result, industry has adopted both existing and new tools to deal with GSD. These tools have features that make them suitable as regards reducing temporal, geographic and socio-cultural distance (Portillo-Rodríguez et al. 2010).

Lanubile et al. (2013) published a survey on the key technologies and tools that support group awareness and collaboration in GSD projects. The systematic literature review (SLR) by da Silva et al. (2010) collected 30 challenges regarding SPM in distributed software development from 54 studies published between 1998 and 2009. Jiménez et al. (2009) identified ten challenges and proposed a continuous improvement model to counter these challenges. In these two studies, a lack of group awareness, communication, coordination and collaboration are considered to be key factors in the complexity of SPM activities in a highly distributed context. Research is still ongoing to discover factors weighing in the failure of GSD projects. Two studies conducted in 2016 (Niazi et al. 2016a, b) aim to identify challenges that might undermine the success of such projects. These studies were carried out by the means of an SLR and were validated using data retrieved from questionnaire based survey. Their results indicate that GSD projects still suffer from a lack of communication and coordination between stakeholders.

In this paper we report a systematic mapping study (SMS) on the tools used for SPM in the context of GSD. Its purpose is to identify these tools, to classify them using an international standard [ISO/IEC/IEEE 12207 “Systems & Software Engineering—Software Lifecycle Process” (2008)] and to show how they support practitioners’ activities. The information provided by this study includes a list of tools and their attributes, such as: License type, technology type (framework, stand alone tool, plug-in$\ldots$) and whether they foster communication, coordination and cooperation between different stakeholders. This may prove useful for software project managers who have to deal with a distributed environment, helping them choose between the variety of tools that are intended to support their activities. Both tool builders and researchers can also identify prominent publication sources for relevant studies and areas of SPM in which the least number of tools is available, thus paving the way for the development of new tools.

The remainder of this paper is organized as follows: “Related work” section identifies related work concerning the topic of the SMS. “Mapping process” section presents the systematic mapping, including its planning, conduction and analysis. In “Results and discussion” section, the results and findings of the study are shown and discussed and the implications for researchers and practitioners are then described. “Threats to validity” section states the limitations of the review. Finally, “Conclusions and future work” section presents the conclusions of this SMS and possible future work.

## Related work

Early research on the subject was conducted by da Silva et al. (2010) in an SLR carried out in 2010, in which researchers were able to list 24 tools intended for SPM use in a highly distributed environment. The goal of this study was to identify the challenges confronted by project managers in this context, best practices that overcome these difficulties and both the tools and models that support these practices. An evidence-based project management improvement model was ultimately presented. This model aims to provide practitioners and researchers with support as regards attaining a better understanding of the landscape of GSD project challenges and devise more effective solutions to improve project management in a distributed setting. In the same year, Portillo-Rodríguez et al. (2010) published a survey of 35 tools classified with the use of the processes presented in the ISO/IEC 12207 standard (2008). A matching between these tools, their features and the distance factor they reduce was presented.

In an SMS published in 2012, Portillo-Rodríguez et al. (2012) collected 132 tools intended for use in the GSD realm and classified them by considering the areas defined in the SWEBOK (Abran et al. 2001). According to their study, 21 tools are used to engineer project management, while the others are more oriented toward the technical process of software development. The same authors also presented a list of empirically validated tools, one of their findings regarding this question being that only 25.8% of the tools listed in their study have been evaluated in a distributed environment.

Tell and Ali Babar (2012) tackle the same subject as (Portillo-Rodríguez et al. 2012) more extensively and classify the tools using various categorizations. In their mapping study, they were able to identify and classify 412 tools intended for the GSD domain extracted from a set of 182 primary studies. The researchers used three classifications schemes, the first being the technology type of the tools, the second being which of the 3C (communication, coordination, cooperation) collaboration model dimensions are supported and the last being which of the software development activities, the tool intends to support. The 3C collaboration model has been proposed by Ellis et al. (1991) and adopted by Fuks et al. (2008). It advocates the analysis, representation, and development of groupware by means of the interplay between the 3Cs, namely, Communication, Coordination, and Cooperation. The model became widely accepted for analyzing tools employed to support computer-mediated interactions (Tell and Ali Babar 2012) and has also been used as a classification scheme in this SMS.

Portillo-Rodríguez et al. (2010) and Tell and Ali Babar (2012) both published a set of tools intended for GSD use as part of an SMS study. These two studies were conducted at nearly the same time while researchers were unaware of the other study for most of their research and can give a useful insight on how the method used can alter the results. The study from Tell and Ali Babar (2012) offers a list of 412 tools while the study from Portillo-Rodríguez et al. (2010) offers a list of only 132. Tell and Ali Babar (2012) aware at the end of the other study discuss these differences and claim it to be essentially due to the search terms used and digital libraries queried. Another difference is that some tools have been classified differently in these studies. These tiny differences occurred not only because they used different classification methods but also because they attributed each tool to a unique class while some tools tend to have more than just one use and can be attributed as such to different classes. Associating each tool to a unique class can hinder the preciseness of the results produced and has been avoided in this SMS.

To the best of our knowledge, no systematic mapping or review of the tools available in GSD has been performed to date with a focus on SPM activities and processes. As stated above, da Silva et al. (2010) proposed a model for SPM in a GSD context, but they did not focus on the tools used nor classify them according to a widely known standard. Furthermore, although Portillo-Rodríguez et al. (2012) and Tell and Ali Babar (2012) present exhaustive lists of the GSD tools that are available, in these studies, project management solely comprises project planning, while other areas such as knowledge management and configuration are not considered as part of engineering project management. In this study, we aim to aggregate and update lists of tools used in GSD, provided by selected secondary studies, while focusing on SPM processes.

## Mapping process

SMSs are designed to provide a classified scheme of a broad research area, and to categorize existing research evidence on a topic and its subsequent results. These results can identify gaps in research, and an SLR can then be used to study these gaps in greater depth. In this section, the research questions (RQs) of this study are first laid down and the search string used to query the digital libraries is constructed based on the PICO method. Afterwards, digital libraries used are specified, Inclusion and Exclusion criteria are defined and a quality assessment method is established. Finally the data extraction method is indicated for each RQ. The protocol of this SMS is based on the recommendations of Kitchenham and Charters (2007), and the method used is presented in the following subsections.

### Research questions

The RQs aim to classify the tools available in terms of features and type and to assess their use in the software industry. The RQs and their motivations are displayed in Table 1.

**Table 1:** RQs and main motivations

Id	Research question	Main motivation
RQ1	Which publication channels are the main targets for SPM tools for GSD research?	To identify where relevant research of the topic can be found and targets for the publication of future studies
RQ2	How has the paper publication frequency on the topic of SPM tools for GSD changed over time?	To identify existing publication trends
RQ3	What are the research types of the selected papers?	To identify research types in literature related to GSD tools
RQ4	What are the research approaches of the selected papers?	To identify research approaches and whether validation of SPM tools for GSD has been published in literature
RQ5	What are the SPM tools used in the GSD context? What are their main features?	To support practitioners with information regarding SPM tools intended for GSD
RQ6	Are all SPM activities properly supported by SPM tools for GSD?	To identify which SPM activities researchers are more interested in and what activities require more investigation
RQ7	On which of the 3C dimensions (Communication,coordination and cooperation) the SPM tools used in GSD focus?	To identify to what extent SPM tools for GSD support group interactions according to 3C model

For the purpose of this study, SPM is defined as the project processes of the ISO/IEC/IEEE 12207 “Systems and Software Engineering—Software Lifecycle Process” (2008). Project processes include project management processes that are used to establish and evolve project plans, to assess actual achievement and progress against the plans and to control execution of the project through to fulfillment, along with project support processes that support specialized management (2008). Individual processes are defined in “Data extraction and synthesis” section—RQ6.

### Search strategy

In order to answer the RQs, a search was conducted using a search string composed of keywords relevant to this study and applied to a number of academic electronic libraries and search engines.

#### Search string

Following the guidelines of Kitchenham and Charters (2007) using the PICO method (population, intervention, comparison, outcome), the keywords initially identified from the RQs are:

- Population: *Global software development*.
- Intervention: *Software project management*.
- Outcome: *Tool*.

Comparison is not relevant in this study, since it is an exploratory study. The Intervention keyword has proven to be limiting the group of studies targeted by this SMS. The reason being that most papers do not talk specifically about SPM but rather SPM processes (e.g. planning, version control), consequently, the intervention keyword has been dropped.

The search terms that have similar meanings are organized into groups. Combined terms are obtained using the OR logical operator between search terms in the same group. The final search string is obtained using the AND logical operator between combined terms of different groups. The search string used is: [(global OR distributed OR outsourcing OR located OR offshore OR collaborative) AND (software) AND (development OR engineering OR improvement OR project) OR GSD OR DSD OR GSE OR CSE] AND (tool OR technology).

The search terms used have been inspired from similar research (da Silva et al. 2010; Portillo-Rodríguez et al. 2012) and the authors suggestions.

#### Literature resources

Automatic searches using the specified search string has been performed in the following digital databases in January 2015:

- Digital libraries
  - IEEE Xplore (2016).
  - ScienceDirect (2016).
  - Association for computing machinery (ACM) (2016).
- Digital search engines
  - Google Scholar (2016).
  - Digital bibliography and library project (DBLP) (2016).

The digital libraries used in this SMS have been selected by using previous SLRs and SMSs in the same field and with a similar scope as a basis. In studies (da Silva et al. 2010; Portillo-Rodríguez et al. 2012; Jiménez et al. 2009; Costa et al. 2010), researchers have unfailingly used IEEE Xplore, ACM and ScienceDirect libraries and the studies selected from these digital libraries represent 79, 92, 83 and 80% respectively of the total number of studies selected in each review. Two additional search engines (Google Scholar and DBLP) were also used in order to include more results.

The impossibility of performing the search in all the digital databases and search engines using the same method signified that a specific configuration of the search string was used with each search engine. Command search queries used in each digital library and search engine is available in Appendix 3.

### Study selection procedure

Candidate studies from the automated searches were reviewed by two authors separately, who were asked to determine the relevancy of papers based on their title and abstract. A paper was accepted if both researchers agreed that the study was relevant; it was refused if both researchers agreed that the paper was irrelevant. Those papers upon which the researchers could not reach an agreement were reviewed in a second phase, during which the researchers resolved their disagreements in a meeting in which they exchanged their ideas on the content of the papers and their relevancy after studying the full text of the article.

In the case of a journal article extending a conference paper, both papers are selected as long as they pass the selection procedure. During the study selection procedure, relevant studies were identified using the inclusion and exclusion criteria cited here after:

#### Inclusion criteria

1. The paper studied one or several tools that support SPM activities in a GSD context.

#### Exclusion criteria

1. The paper studied SPM techniques (without tools) used in GSD.
2. The paper was a workshop summary.
3. Paper was not in English.

The exclusion criteria were applied using “OR” logical operator between them.

### Tool identification and classification procedure

Although some tools can be thought not to be related to this research, further investigation of their use in industry and their features proved otherwise. A tool like “Eclipse help system” is primarily a help system for an IDE and would commonly be considered to be outside of this research’s scope. Nonetheless, it allows users to create and modify project documentation, which can then be used by other stakeholders. This feature allows knowledge to be created and shared. This tool can then be included in the group of tools that support the IM process. “Social software” can also be a source of confusion as they are frequently used inside and outside work context, but recent studies indicate that tools such as Skype, Twitter, etc., have been widely used in the GSD industry to provide additional communication channels (Niazi et al. 2015; Giuffrida and Dittrich 2013).

Information necessary to classify the tools was retrieved primarily from the selected studies. In case information is incomplete or missing, it was retrieved from the tool builder website or generic information on the Internet. The first author was tasked with classifying the tools using available information, the remaining authors were tasked with reviewing each a section of the final list of tools in order to cover the whole list. In case of a disagreement, the authors held a meeting where they exchanged their ideas based on viable information until an agreement was reached.

### Quality assessment process

Each of the primary studies selected was assessed to ascertain its quality using four criteria inspired by previous studies (Ouhbi et al. 2015; Fernandez et al. 2011):

- *QA1* The main focus of the study is SPM tools used in a GSD context (yes +1/partially +0).
  Some papers may present a discussion about our research subject but only in a secondary manner, while the main topic of the paper is different. We consider that these papers are not as interesting for our SMS as those which are entirely dedicated to discussing SPM tools in a GSD context.
- *QA2* The study explicitly presents tools with which to support GSD project management activity (yes +1/no +0).
  Some papers may only present guidelines or recommendations as to how to use or develop tools. These papers might be interesting for our discussion but our main research goal is to list all available tools. The paper achieves the full score if a new tool is presented or an existing tool is assessed.
- *QA3* The study uses empirical results for argumentation (yes +1/no +0). The results and conclusions of the study are strengthened by empirical evidence and it provides important and reliable information about future research and practice (Šmite et al. 2010).
- *QA4* The study has been published in a recognized and stable publication source. This rating is based on the 2013 journal citations report (JCR) (2013) for journals and the computer science conference ranking in Computing Research and Education (CORE) 2013 Conference Rankings (2013).
  - Conf: (CORE A* +2/CORE A +1.5/CORE B +1/CORE C +0.5).
  - Journal: (Q1 +2/Q2 +1.5/Q3 +1/Q4 +0.5).
  - No ranking: +0.

JCR uses the impact factor to rank journals by their field of interest. Those ranked in the first quartile are called Q1 journals. Those in the second, third and last are respectively called Q2, Q3, Q4 journals. CORE2013 uses the following ranking categories, derived primarily from earlier CORE ranking exercises: CORE A* (flagship conference), CORE A (excellent conference), CORE B (good conference), CORE C (other ranked conference).

Although there is a general opinion that journal papers are better than conference papers, Bowyer (2012) is the opinion that conferences and journals are different by nature and cannot be compared; we also agree with this statement, since both contribute to the dissemination of knowledge in their own way. The quality classification scheme of the selected studies is based on a global unsupervised discretization method (Dougherty et al. 1995), a variation of the equal width interval binning in which the upper and lower bins are shorter than others with the intention of discriminating between extreme scores. The classification scheme is presented in Table 2.

**Table 2:** Quality classification scheme

Quality level	Corresponding score
Excellent	Score = 5
High	Score $\in$ {3.5, 4, 4.5}
Medium	Score $\in$ {2, 2.5, 3}
Low	Score $\in$ {0.5, 1, 1.5}
Very low	Score = 0

### Data extraction and synthesis

The data needed to answer the RQs in Table 1 were extracted by exploring the full text of each selected article. A spreadsheet was used to store the data concerning each article whose structure is presented in Table 3. Another spreadsheet was used to extract data concerning tools, which is presented in Table 4. The strategy is hereafter explained for each RQ:

- *RQ1* The publication source and channel for each paper is listed and the aggregated results will be presented.
- *RQ2* The publication year for each paper is listed, and the aggregated result will provide an overview of the number of related articles per year.
- *RQ3* The research type for each paper is classified into one of the following categories (Brereton et al. 2007):
  - *Evaluation research* Existing SPM tools are implemented in a GSD context and an evaluation or a validation of these tools is conducted.
  - *Solution proposal* An SPM tool designed for GSD is proposed. This solution may be a new tool or a significant extension of an existing tool. The potential benefits and the applicability of the solution could be shown with an empirical study or a good argumentation.
  - *Review* An analysis of existing literature concerning SPM tools in a GSD context.
  - *Other* Any other research type not listed above (e.g. experience report, opinion paper, etc.).
- *RQ4* The research approach for each paper is classified into one of the following categories (Condori-Fernandez et al. 2009):
  - *Case study* An empirical inquiry that investigates a tool within its real-life context.
  - *Survey* A method for collecting quantitative information concerning a tool (e.g. a questionnaire).
  - *Experiment* An empirical method applied under controlled conditions.
  - *Non empirical* Non empirical research approaches or a theoretical evaluation of a tool.
  - *Other* Any approach not listed above.
- *RQ5* Each of the tools proposed or reviewed in any of the selected papers is listed in order to create an exhaustive set of the tools proposed for SPM activity in literature. We have differentiated between the technologies by adopting the classification proposed in Tell and Ali Babar (2012):
  - *Standalone tool (SAT)* an independent software application fulfilling a specific design intent.
  - *Framework* a “semi-complete” application that provides an integrated set of domain-specific structures and functionality.
  - *Environment* a development environment that comprises a set of processes and programming tools used to create software products. Moreover, an integrated development environment is a subset of this group, which identifies a development environment that has a unified interface.
  - *Platform* a set of generic components that form a common structure, from which a set of derivative products can be developed.
  - *Plug-in* a software component that interacts with an existing software application through the use of well defined application programming interfaces (APIs), often designed to enhance it by adding new functionalities.
  RQ5 also aims to classify tools on the basis of their type of license, and we have adopted the classification used in Portillo-Rodríguez et al. (2012):
  - *Commercial* Tools whose license can be obtained by means of payment, although a free trial period may be offered.
  - *Free* Tools whose license can be obtained without payment. In this license type we include licenses such as Apache License, general public license (GPL), etc.
  - *Research* Tools or prototypes developed by research groups which are not freely or commercially available.
- *RQ6* We have adopted the ISO/IEC/IEEE 12207 “Systems and Software Engineering—Software Lifecycle Process” standard for the classification of the activities related to SPM. The classification of the listed tools will be based on the project processes of (2016). A tool may therefore be focused on one or more SPM processes. The processes have been defined on the basisof (2016):
  - *Project planning (PP)* The purpose of the PP process is to produce and communicate effective and workable project plans. This process determines the scope of the project management and technical activities, identifies process outputs, project tasks and deliverables, establishes schedules during which the project task will be conducted, including achievement criteria, and states which resources are required to accomplish project tasks.
  - *Project assessment and control (PA)* The purpose of the PA process is to determine the status of the project and ensure that the project performs according to plans and schedules, and within projected budgets, and that it satisfies technical objectives. This process includes the redirection of the project activities, as appropriate, in order to correct identified deviations and variations from other project management or technical processes. Redirection may include replanning as appropriate.
  - *Decision management (DM)* The purpose of the DM process is to select the most beneficial course of project action when alternatives exist. This process responds to a request for a decision encountered during the system life cycle, whatever its nature or source, in order to reach specified, desirable or optimized outcomes. Alternative actions are analyzed and a course of action is selected and directed. Decisions and their rationale are recorded to support future decision-making.
  - *Risk management (RM)* The purpose of the RM process is to identify, analyze, treat and monitor the risks continuously. The RM process is a continuous process by which to systematically address risk throughout the life cycle of a system or software product or service. It can be applied to risks related to the acquisition, development, maintenance or operation of a system.
  - *Configuration management (CM)* The purpose of the CM process is to establish and maintain the integrity of all identified outputs of a project or process and make them available to the parties concerned.
  - *Information management (IM)* The purpose of the IM process is to provide relevant, timely, complete, valid and, if required, confidential information to designated parties during and, as appropriate, after the system life cycle. This process generates, collects, transforms, retains, retrieves, disseminates and disposes of information. It manages designated information, including technical, project, organizational, agreement and user information.
  - *Measurement (Me)* The purpose of the Measurement process is to collect, analyze, and report data relating to the products developed and processes implemented within the organizational unit, to support the effective management of the processes, and to objectively demonstrate the quality of the products.
- *RQ7* This question aims to identify which of the 3C are supported by the listed tools. The classification used is inspired from a systematic review of awareness support in GSD carried out by Steinmacher et al. (2010) where the researchers presented an investigation of the role of the limitations of awareness support in the success of GSD projects based on the 3C model:
  - *Communication* When the tool brings improvements to the way in which messages and information are exchanged among people, reducing gaps, ambiguity and the effort needed to understand, establish, and continue a conversation.
  - *Coordination* When the tool focuses on providing people with support in order for them to manage themselves by checking and alerting them to the activities, resources and tasks performed by other people that may influence their work.
  - *Cooperation* When the tool aims to bring improvements to the shared space or the way in which users synchronously or asynchronously interact with shared artifacts.

**Table 3:** Article data extraction form

Study ID	Authors	Pub. title	Pub. source	Year	Type	Approach	Tool
**	**	**	RQ1	RQ2	RQ3	RQ4	RQ5

**Table 4:** Tool data extraction form

Study ID	Tool	License	Type	SPM process	3C model focus
**	RQ5	RQ5	RQ5	RQ6	RQ7

## Results and discussion

**Fig. 1:**
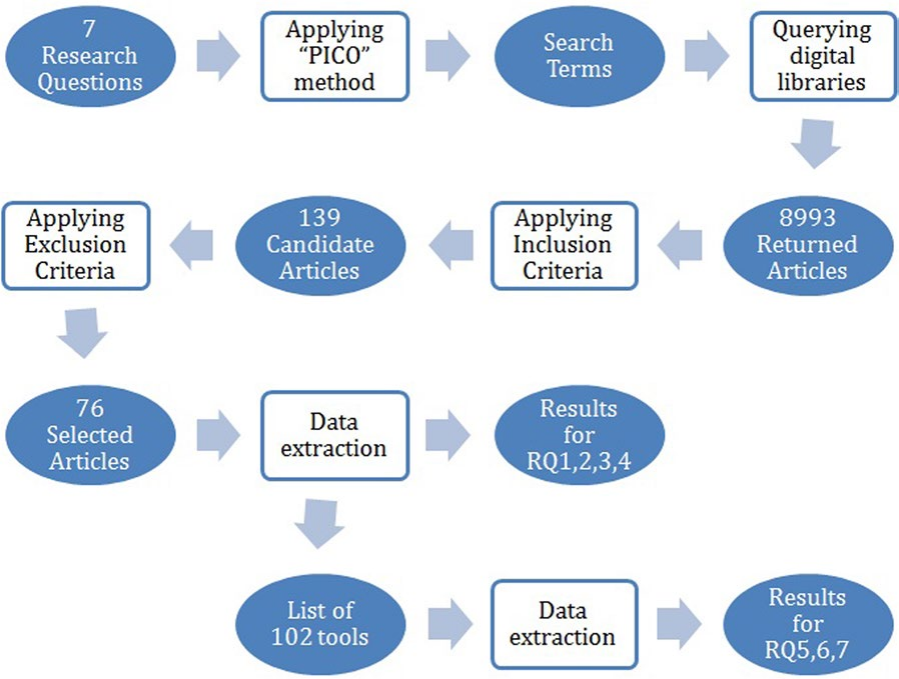
Summary of the mapping processes

In this section, results for the selection procedure and quality assessment for selected articles in this SMS are presented. Then, results for the data extraction are exposed, analyzed and discussed for each RQ. Finally, implications and suggestions for researchers and practitioners are given. Figure 1 summarizes the mapping between this SMS’s operations and products.

### Selected studies

**Fig. 2:**
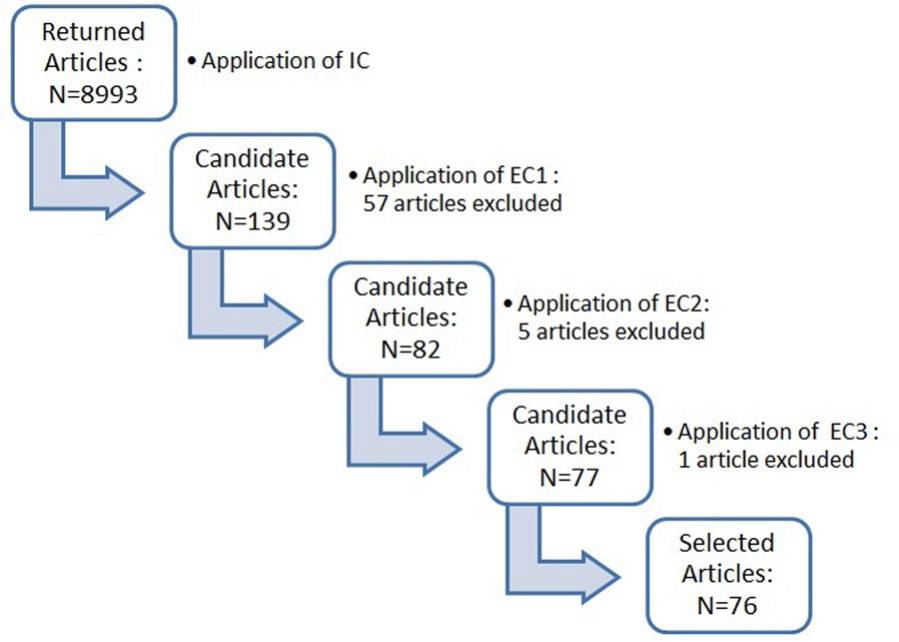
Study selection results

The search string that was applied to the different digital libraries and the search engines returned a high number of results (8993). Based on their title, keywords, abstract and perhaps the full text, the researchers tasked with the study selection process included those articles that might possibly answer the RQs presented in Table 1. After completing the process explained in “Study selection procedure” section, 76 articles were selected. Thirteen articles were judged differently in the first phase but an agreement was reached for these articles on the second phase. The Cohen’s Kappa coefficient was used to calculate the interrater agreement between the two authors in their evaluation. The Kappa coefficient was 0.808 which according to Landis and Koch (1977), indicates a strong agreement between the two assessments. Figure 2 shows the results of the selection process, where “N” is the number of the remaining identified articles at each stage of the selection process.

### Quality assessment

The rules shown in “Quality assessment process” section were used to conduct a quality assessment of the selected studies. The score obtained by each study is not systematically a sign of their quality, since studies with lower scores have a different research scope but are still useful as regards answering the RQs.

Kitchenham and Charters (2007) specifies that some researchers use quality assessment as selection criteria in their SLRs, but it is not mandatory. Following this rule, no studies have been discarded during the quality assessment process. The aim of this SMS is to gather an exhaustive list of the available SPM tools used in GSD, and such tools may be found even in those studies with lower score.

The main motivation of this quality assessment is to provide an overview of the usefulness of the selected papers in this SMS. Results show that 75% have an average score of 2.5 points or higher. Table 5 provides information about the total score of the selected articles. Detailed scores for each of the studies selected are available in Table 9 in Appendix 1.

**Table 5:** Articles quality level and percentage

Quality level	Number	%
Excellent	3	3.95
High	28	36.85
Medium	33	43.4
Low	9	11.85
Very low	3	3.95

Table 6 shows the number of articles based on the ranking of the conference or journal in which they were published.

**Table 6:** Articles by their journal or conference rank

Journals		Conferences	
Q1	8	CORE A*	2
Q2	4	CORE A	8
Q3	2	CORE B	6
Q4	0	CORE C	33
No ranking	1	No ranking	12

### RQ1: Source and channel of publications

As shown in Table 7, 38% of the selected papers, were presented at the International Conference on Global Software Engineering and its related workshops. This result clearly shows that the ICGSE conference is the main publication source for our topic. The ranking of the ICGSE is CORE C based on the latest CORE 2013 and it is the premier conference devoted to GSD. It has attracted the attention of several renown authors. For example, the works by Lanubile et al. (2013) and Prikladnicki et al. (2012) are derived from the analysis of the articles published in this conference. With regard to journals, IEEE Software is the journal with most publications focusing on SPM tools in GSD. It is ranked Q1 in JCR and four of the articles selected have been published in it.

**Table 7:** Publication source and channel

Pub. Source	Pub. channel	Articles	Number
IEEE International Conference on Global Software Engineering, ICGSE	Conference	Ali et al. (2010), Aranda et al. (2006), Cataldo et al. (2009), Clear (2009), Dullemond and van Gameren (2012), Dullemond et al. (2009, 2010), Gupta and Fernandez (2011), Jaanu et al. (2012), Lamersdorf and Munch (2009), Liukkunen et al. (2010), Martignoni (2009), Mullick et al. (2006), Niinimaki and Lassenius (2008), Niinimaki et al. (2010), Paulish (2006), Portillo-Rodríguez et al. (2010), Prause et al. (2010), Salger et al. (2010), da Silva et al. (2010), Sinha et al. (2007), Spanjers et al. (2006), Dullemond and van Gameren (2013), Costa and Murta (2013)	24
International Conference on Global Software Engineering Workshops, ICGSEW	Workshop	Beecham et al. (2012), Garrido et al. (2012), Giuffrida and Dittrich (2011), Pesola et al. (2011), Tell and Ali Babar (2011b)	5
IEEE Computer Society’s IEEE Software	Journal	Prikladnicki et al. (2012), Sinha et al. (2006), Lanubile et al. (2013), Lanubile et al. (2010)	4
Information and Software Technology	Journal	Portillo-Rodríguez et al. (2012), Giuffrida and Dittrich (2013), Sakthivel (2005), Al-Ani et al. (2014)	4
International Conference on Software Engineering Advances, ICSEA	Conference	Geisser et al. (2007), Eskeli et al. (2011), Scharff et al. (2010)	3
Portland International Center for Management of Engineering and Technology Conferences, PICMET	Conference	Samoilenko and Nahar (2012a, b), Wesslin et al. (2011)	3
Collaboration Researchers International Working Group Conferences, CRIWG	Conference	Aranda et al. (2011), Monasor et al. (2010a)	2
International Conference on Evaluation and Assessment in Software Engineering, EASE	Conference	Costa et al. (2010), Winkler et al. (2010)	2
International Conference on Software Engineering, ICSE	Conference	Bowen and Maurer (2002), Ramasubbu and Balan (2012)	2
Other	Conference	Cook et al. (2005), Tell and Ali Babar (2011a), Mak and Kruchten (2006), Lam and Maheshwari (2001), Gorton et al. (1997b), Aranda et al. (2008), Simmons and Ma (2006), Herring and Rees (2001), Monasor et al. (2010b), Thissen et al. (2007), Murdoch and Astley (2004), Wu (2012), Miyamoto et al. (2012), Goedicke et al. (2000), van Hillegersberg and Herrera (2007), Chubov and Droujkov (2007), Vathsavayi et al. (2014)	17
Other	Journal	Jiménez et al. (2009), Gorton et al. (1997a), Persson et al. (2009), Treude and Storey (2012), Palacio et al. (2011), Portillo-Rodríguez et al. (2014)	6
Other	Workshop	Wang et al. (2012), Simmons (2003), de Souza and Fonseca (2007), Surjaputra and Maheshwari (1999)	4

The main publication target for articles related to the use of SPM tools in a GSD context would generally appear to be conferences, as 70% of the selected articles have been published via this channel. Table 9 in Appendix 1 provides more details on the selected articles per publication source and channel.

### RQ2: Publication distribution per year

**Fig. 3:**
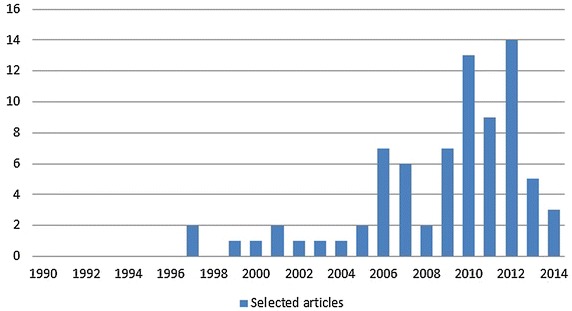
Number of publication per year

Figure 3 shows the number of primary studies per publication year. There has been a sizable increase in publications since 2006. This year corresponds to the first ICGSE conference and the increasing academic concern as regards studying the effect of globalization on the software industry; the same conclusion was reached by da Silva et al. (2010).

### RQ3: Research type

Of the selected articles, 46% are solution proposals that have been derived solely from academic research. This prevalence hints that there may be a lack of existing tools with which to mitigate the effect of globalization on the software industry. In addition, 25% of the selected papers are evaluation research. Reviews represent 15% of the selected papers while another 14% represent the “Other” category, comprising opinion reports, experience papers and so on.

**Fig. 4:**
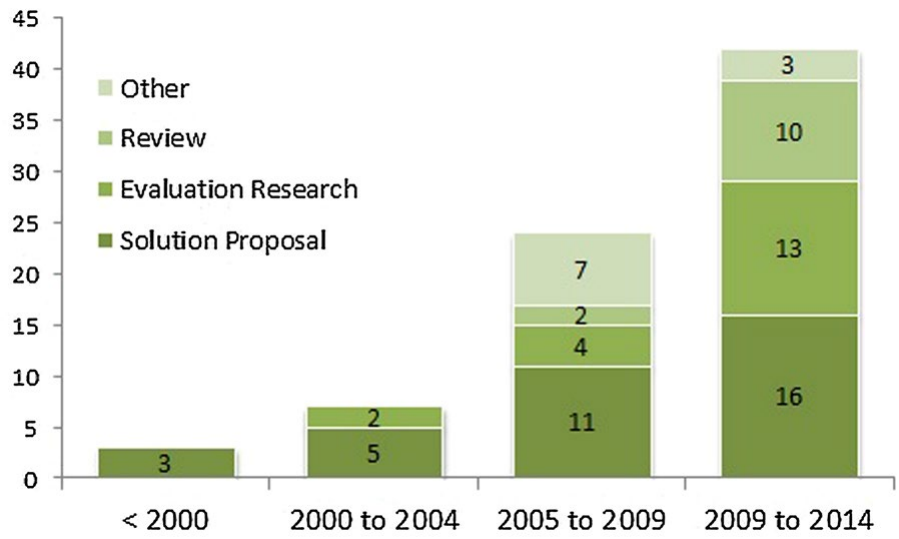
Number of papers by research type per time period

According to the data shown in Fig. 4, the number of evaluation research papers is low in comparison to the number of solution proposals up until 2009. SPM in GSD is still a relatively new subject with different areas that have yet to be researched and explored. This has pushed researchers to produce several new tools that are intended for use in the GSD context (Lam and Maheshwari 2001; Sinha et al. 2007; Simmons and Ma 2006; Bowen and Maurer 2002). However, in order to carry out further research on this subject, empirical data was needed to assess the use and benefits of different tools used by project managers in GSD projects. This has led to a sizable increase of evaluation research and reviews in comparison to solution proposals.

These results can be explained using the Redwine & Riddle maturation model (Shaw 2002). This model stipulates that there is a trend indicating that technologies take 15–20 years to evolve from concept formulation to the point at which they are ready for popularization. The first 10 years of this evolution are spent investigating basic ideas and concepts, then developing a research community that converges on a compatible set of ideas and finally making a preliminary use of the technology and generalizing the approach.

### RQ4: Research approach

The existing literature offers a relatively high number of solution proposal studies to support the management activity of GSD projects, and half of them have no empirical data to support their usefulness.

**Fig. 5:**
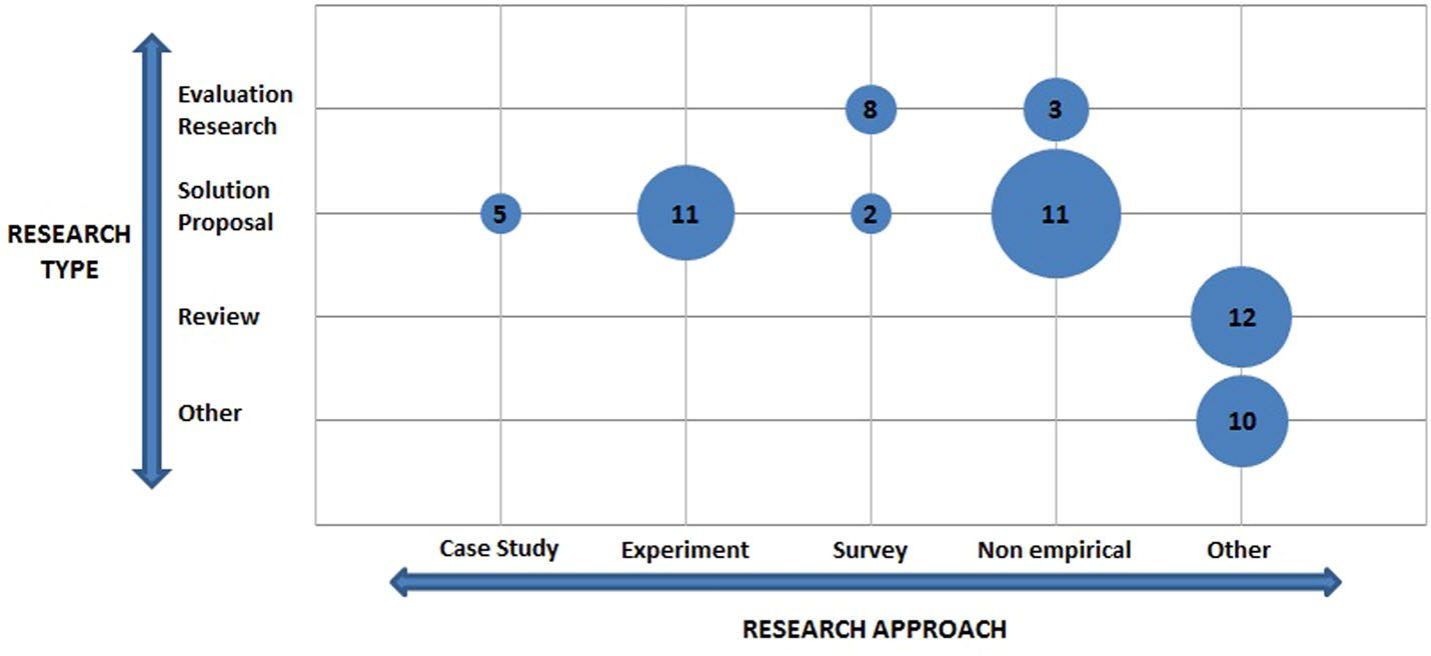
Research types and approaches

As shown in Fig. 5, 57% (20 of 35) of the solution proposal studies included in our SMS are not validated empirically, while 31% (11 of 35) are validated through experiments. It has been noted that most of these tools are academic, showing a lack of collaboration between industry and researchers in this field. There have simultaneously been very few evaluations of the existing tools used in industry. Only eight evaluation papers are based on industrial case studies.

In our opinion, this situation may have been caused by two factors:

1. SPM experiments in GSD are difficult to conduct. GSD settings imply that the subjects of the experiment must be from different geographic locations, different time zones and different cultures. This can complicate the empirical evaluation of tools in industrial environments (Šmite et al. 2010).
2. Although some examples can be found in literature (Habra et al. 2008; Cuadrado et al. 2014), software companies are unlikely to collaborate with researchers in project management because they keep their research and tools confidential.

### RQ5: SPM tools used in GSD

**Fig. 6:**
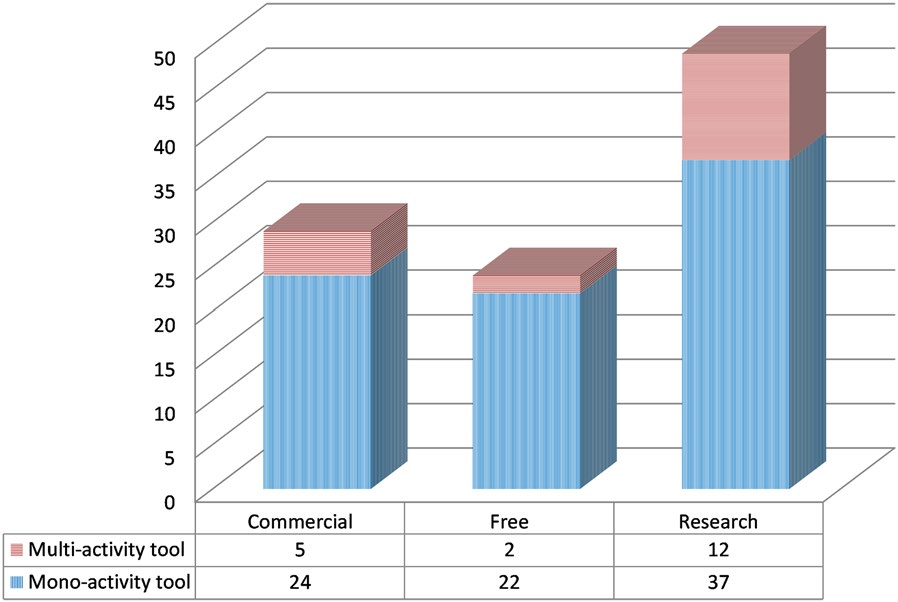
Tools by license and number of areas covered

After synthesizing the data obtained from the selected studies, a list of 102 tools was compiled. Figure 6 displays the number of the tools retrieved from literature based on their type of license and whether they focus on one or several SPM processes. Results show that 48% of these tools are the fruit of academic research which seems logical considering that most of the literature reviewed is research related, 24.5% of the tools are free and open source software (FOSS) while the remaining 28.5% are commercial tools.

As shown in Fig. 6, tools focusing on a specific process (mono-activity tool) are dominant in comparison with tools focusing on several processes (multi-activity tool). For example, tools like Travis (Geisser et al. 2007), DPMTool (Garrido et al. 2012) and Microsoft Office Communicator (Niinimaki et al. 2010) focus on a particular process while other tools focus on several activities (see Table 12 in Appendix 2). Another example of a single activity tool is Atlassian JIRA (Prikladnicki et al. 2012; Lanubile et al. 2010) which is considered to be an issue tracker without any other relevant features, thus focusing only on the PA process. IBM Rational Team Concert (Scharff et al. 2010; Treude and Storey 2012; Wang et al. 2012), meanwhile, has PP, artifact management and messaging features. In this case, it is focused on PP, CM and IM processes.

We noted that 24% of research, 17% of commercial and 8% of free tools focus on more than one SPM process. Researchers tend to create tools that cover most of the development lifecycle process. Tools like: Enabler Framework (Sinha et al. 2007), GWSE (Gorton et al. 1997a), Milos ASE (Bowen and Maurer 2002), NextMove (Mak and Kruchten 2006), PAMPA2 (Simmons 2003), PSW (Eskeli et al. 2011) and SEES (Simmons and Ma 2006) fall into this category (see Table 12 in Appendix 2).

**Fig. 7:**
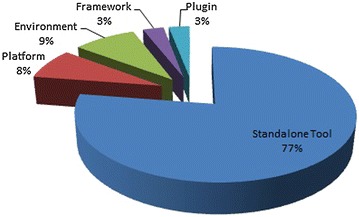
Percentage of tools by type

The majority of the tools listed in this study (79 out of 102) are SATs that are intended to satisfy a specific design. However, the use of SATs increases context switches, which can be a source of frustration (Sengupta et al. 2006). The second largest category is environment, of which there are 9. The third largest category is platforms with eight tools. Note that platforms are dominated by commercial solutions (6 out of 8). This can be explained by the effort required to develop platforms in terms of time and human resources that can barely be afforded by researchers (Sengupta et al. 2006). Figure 7 shows the percentage for each category. The extensive list of the tools available that have been obtained from the literature review is provided in Table 12 in Appendix 2.

During the data extraction process, two entities were found to be behind several of the studies selected in this SMS. First, the Alarcos Research Group at the University of Castilla-La Mancha in Spain authored 11 studies regarding tools used in SPM for GSD projects (Portillo-Rodríguez et al. 2010, 2012, 2014; Jiménez et al. 2009; Garrido et al. 2012; Lanubile et al. 2010; Aranda et al. 2006, 2011; Monasor et al. 2010a, b; Aranda et al. 2008; Palacio et al. 2011). Second, the IBM research lab, which involves researchers from India, Japan and the USA, published three papers (Sinha et al. 2006, 2007; Miyamoto et al. 2012). IBM is one of the largest developers of solutions for SPM in GSD projects with tools like IBM Lotus Notes, IBM Lotus Quickr, IBM Lotus Sametime, IBM Rational ClearCase, IBM Rational Team Concert.

Considering that globalization affects countries all over the world, presenting a ratio of the selected studies per country may be enlightening in the context of this study. This ratio was obtained by using the following method: each selected study was awarded one point. This point was then divided equally between the researchers involved in the study. Finally, each country accumulated points for researchers based on the location of their affiliated entities. As an example, study (Thissen et al. 2007) was carried out by two researchers from the USA, one from Australia and another from India. The USA therefore obtains 0.5 of a point, Australia 0.25 and India 0.25. These results are presented in Table 8.

**Table 8:** Studies ratio per country

Country	%	Country	%
USA	12.77	Brazil	7.17
Finland	12.60	Denmark	6.58
Spain	11.59	The Netherlands	6.25
Germany	9.74	India	4.71
Australia	8.22	Others	20.37

In this perspective it will be noted that the USA has the highest percentage followed closely by Finland and that European countries are heavily involved in research on this particular topic. Nonetheless, typically outsourced emerging countries like Brazil and India (Javalgi et al. 2009) are also contributing to this research line.

### RQ6: SPM areas covered by tools

**Fig. 8:**
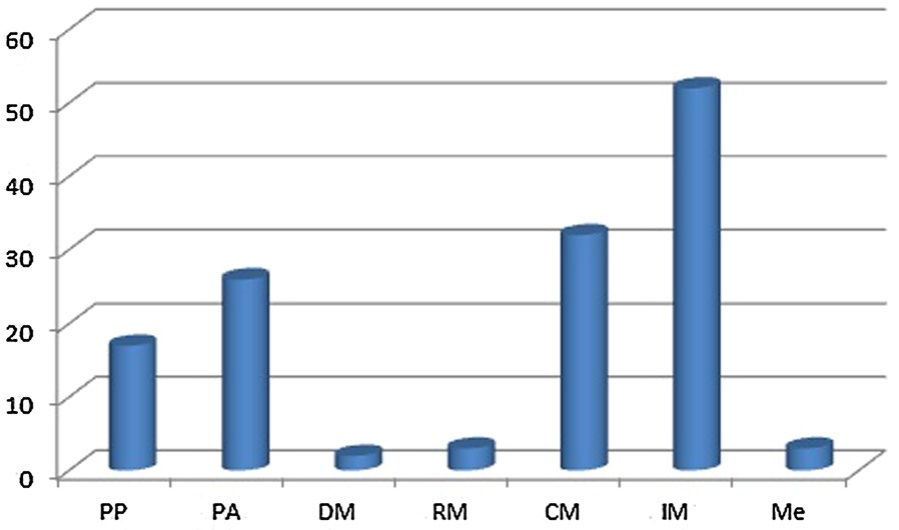
Number of tools covering each SPM area

Figure 8 presents the number of tools per SPM process. It shows a disparity in the number of tools identified between the SPM processes.

The IM process suffers the most from the effect of high distribution (Portillo-Rodríguez et al. 2012), which is considered to be one of the top challenges regarding SPM in a global context (da Silva et al. 2010). This area is covered by 52 of the 102 identified tools—a little bit more than half of them. However, it is important to note that in our classification, and in contrast to other surveys (Portillo-Rodríguez et al. 2010; Giuffrida and Dittrich 2013), we consider that social software and knowledge management tools fall under the scope of the IM process. These tools generally focus on the communication aspect of the 3C Model, since they have integrated features of instant messaging, forums or wikis.

The CM process is covered by 32 tools. They are version control tools which are more focused on collaboration and concerned with the integrity of the project’s outputs (software or documents). Their prominent feature is automatic messaging, which is triggered by new version updates to alert the parties concerned.

PA and PP processes are covered by 26 and 17 tools respectively that enhance collaboration in distributed teams. Eight of these tools propose features that cover both PA and PP processes, namely: ActiveCollab (Lanubile et al. 2010), BaseCamp (Prikladnicki et al. 2012), Enabler Framework (Sinha et al. 2007), GWSE (Gorton et al. 1997a), Issue Player (Portillo-Rodríguez et al. 2012), MILOS ASE (Bowen and Maurer 2002), PSW (Eskeli et al. 2011), Workspace Activity Viewer  (Prikladnicki et al. 2012). However, six of the issue trackers considered in this study fall under the heading of assessment and control processes and are not concerned with planning, thus focusing solely on issue management, such as Atlassian JIRA (Lanubile et al. 2010) and Bugzilla (Lanubile et al. 2010).

DM, RM and measurement processes have very few tools to support their activities when compared to other SPM areas. Only three tools support RM, three tools support measurement and two tools support DM. Researchers should focus more on these areas as they are inadequately supported by proper tools. RM should particularly attract tool builders’ attention. Controlling the risks in software projects in general (Bannerman 2008), and global software projects in particular (Persson et al. 2009), greatly contributes to project success.

According to the Capability Maturity Model Integration version 1.3 (CMMI v1.3) (CMMI Product Team 2010), Me is considered to be a core process of the 2nd maturity level denominated as “Managed” while DM and RM are considered to be core processes of the 3rd maturity level, which is termed as “Defined”. The fact that these areas are not adequately supported by tools might indicate that SPM processes for GSD projects are not yet mature (Šmite et al. 2010). These projects often include ad hoc reactive processes that are unable to anticipate problems.

### RQ7: 3C collaboration model comparison

The tools that have been identified have specific features that manage the challenges that high distribution poses as regards the success of GSD projects. Three key areas have been tackled by GSD tools to support group interaction, namely the 3Cs:

- *Communication* The tools studied integrate a number of features that are intended to reduce the effect of geographical distance and time zone differences on group awareness. These features include instant messaging, forums, e-mail notifications, audio/videoconferencing, and wikis. A total of 49 tools support communication using these features. Lack of communication is considered to be one of the key challenges when managing traditional or agile software development projects in a globally distributed environment (Dullemond et al. 2009). Informal communication in particular is frequently used in agile software development (stand-up meetings, face to face communication, etc.), but is, however, poorly represented. In this regard, Dullemond et al. (2010), Dullemond and van Gameren (2012) have developed a tool named “Communico” that creates a virtual open space in which users can overhear others’ conversations.
- *Coordination* Coordination between project actors is ensured through an improved awareness of team members’ activities. A total of 40 tools support coordination by using e-mail notifications or a dynamic visualization interface of team project members and their respective activities. Tools like ActiveCollab (Portillo-Rodríguez et al. 2010), WorldView (Prikladnicki et al. 2012) and Workspace Activity Viewer (Lanubile et al. 2010) provide an overview of ongoing project activities and give managers an overview of project status at different levels of detail. This information can be used by project managers or developers to enhance coordination and task allocation in a globally distributed software development team.
- *Cooperation* According to Gorton et al. (1997a), the vast majority of the tools used in a collocated development context are designed to support only single-user activity, thus making the exchange of information between users more difficult. This problem is particularly frequent in the CM process in which the project metadata must be shared in a controlled manner. In this SMS, 69 cooperative tools have been identified, which makes cooperation the most prominent feature in SPM tools for GSD. The tools in this category are mainly artifact management or versioning system tools that provide a shared and distributed workspace by using either a centralized or a peer-to-peer (P2P) architecture.

**Fig. 9:**
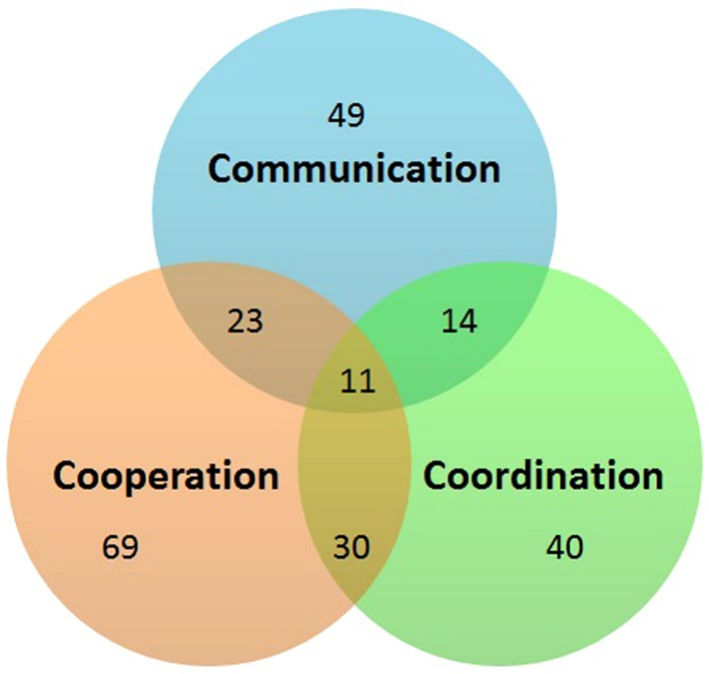
Number of tools according to the 3C collaboration model dimensions

Figure 9 shows how many tools focus on communication, coordination, cooperation or a combination of them. For example, 49 tools have features that focus on communication, and 14 of those also focus on coordination. On the other hand 23 tools focus on communication and cooperation. Finally, eleven tools consider all sides of the 3C model, i.e., communication, coordination and cooperation. See Table 12 in Appendix 2 for more information on this subject.

### Implications for research and practice

The results of this research contribute to GSD in many ways. Firstly, they provide the academic community with a better understanding of the SPM activities and tools used in GSD settings and show gaps in the areas in which tooling is insufficient or inadequate. This information can open up opportunities for future advances by both researchers, who will better understand the landscape of GSD and tackle its challenges, and tool builders, who may wish to provide new tools that are capable of mitigating the effect of globalization on the management of software projects. Secondly, project managers involved in highly distributed projects will have an overview of the existing tools intended to support their activities and identify those which are most likely to meet their needs. The recommendations to researchers and practitioners resulting from the conclusions drawn from this SMS are as follows:

- Practitioners such as tool builders and project managers, along with researchers involved in GSD project management, are eager to know about latest research on the subject. They should view articles published in the proceedings of the ICGSE conference and its affiliated workshops. The latter is the main publication source for studies about SPM in GSD. In the same line, researchers are encouraged to send their articles to this conference.
- The results of this SMS indicate a lack of empirical validation of the various solutions proposed. Only 24.7% of the tools identified in our study have been empirically validated. A similar percentage was obtained in a previous mapping study of GSD tools (Portillo-Rodríguez et al. 2012). Researchers are encouraged to assess their tools using experiments in order to provide qualitative and quantitative data about tool usage. In this case, we encourage geographically separated research groups with socio-cultural differences to collaborate, thus making it easier to simulate GSD experiments whilst simultaneously benefiting from the advantages of global distribution (Prause et al. 2010). We also encourage practitioners to collaborate closely with research groups in order to validate existing tools through case studies or surveys, thus providing useful data which tool builders can use to create tools that are capable of satisfying the demands of GSD project management.
- A number of tools that are, according to literature, intended to support SPM activities in the GSD context have been listed in this study. Practitioners, and particularly project managers, can use this list to select tools that can support their activities. License type and technology type are specified for each tool in order to ease this choice (see Table 12 in Appendix 2).
- Decision management, risk management and measurement processes are not adequately supported by tools when compared to the other SPM processes. Researchers are encouraged to engage in lines of research that may enable the reasons for this disparity to be discovered. Practitioners are encouraged to fill the tooling gaps in these processes.

## Threats to validity

The validity of the study is concerned with the trustworthiness of its results. The conclusions of this research may have been threatened by bias resulting from the researchers’ subjective point of view. The categorization used in Runeson et al. (2012), Wohlin et al. (2012) is adopted to identify the limitations of this SMS. These limitations are classified as follows:

### Construct validity

Construction validity refers to the extent to which the operational measures that are studied really represent what the RQs aim to answer. In an SMS, two factors can be easily identified as a threat to construct validity. One is the research string used, while the other is the digital libraries researched. In this study, we have performed a systematic search using an extensive range of terms to widen our scope of research. The search keywords were proposed by two authors in several iterations to ensure that all relevant literature would be included in the study. Another threat to the construct validity is the choice of digital libraries used. This risk was mitigated by the identification of three digital libraries as the main source of related articles according to existing literature. Two digital search engines were also used to provide additional sources for related articles. A manual search of the reference lists in the selected studies in order to complement the SMS is strongly suggested by Kitchenham and Charters (2007). This process has not been conducted and is considered to be one of the limitations of the construct validity.

### Internal validity

With regard to the internal validity of the study, the classification and the decision to assign a specific tool to a specific area of project management can be judged to be subjective. Similar studies (Portillo-Rodríguez et al. 2012; Tell and Ali Babar 2012) have, for example, classified tools in a different manner and in different fields. In order to decrease this effect, the classification scheme was proposed and the categorization process was carried out by two authors while the others reviewed the final results. Moreover, the steps and activities in this scheme were clearly described to allow the conclusions drawn from the results of this SMS to be reproduced. We have attempted to decrease this threat, by displaying the data retrieved from each of the articles selected in Tables 9, 10 and 12 of Appendices 1 and 2 in order to enable interested readers to check their validity.

### Conclusion validity

Conclusion validity is the degree to which the conclusions we reach about relationships in our data are reasonable and is concerned with the ability to replicate these findings. In an SMS, the threat to conclusion validity is a factor that may lead to an incorrect conclusion being reached about a relationship in the observed data. Bias as regards both selecting and classifying primary studies and tools along with analyzing data may therefore affect the interpretation of the results. In order to mitigate this threat, each step of the selection, extraction and analysis of the data was validated by means of the systematic process and the periodic reviews carried out by the researchers involved in this work. Finally, this SMS has inherited the threats to validity of its primary studies.

### External validity

External validity concerns how far the results of a study can be generalized. In this case, it concerns the external validity of the tools presented. The ratio of academic tools to commercial tools, along with the fact that the selected studies included in this SMS that discuss industrial tools are written by authors who are involved in both research and industrial fields, lead us to believe that our tool list is not exhaustive and that some of the tools used in industry may not have been included. Furthermore, some details about the tools were difficult to obtain, since our tool list was indirectly built, starting from the primary studies and not from the tool’s experience. Nevertheless, the results of this study may serve as a starting point for SPM for GSD researchers and practitioners.

## Conclusions and future work

This paper reports an SMS that explores how tools support SPM activities in GSD. Seventy-six studies were selected and a total of 102 tools were identified in literature and were classified according to the following criteria: license, type, SPM area they support and dimension of 3C collaboration model they focus on. During the process of this SMS, we noticed that although a large number of standalone tools are proposed, fewer tools cover the whole SPM process. A commercial platform and an environment created by international companies have been encountered during research prior to this SMS: IBM Rational and Microsoft Visual Studio. These tools have integrated a number of commercial standalone tools that collaborate and exchange information in a seamless manner, thus ensuring that the project manager’s activities are consistently supported throughout the development lifecycle.

Two studies (Sinha et al. 2007; Eskeli et al. 2011) present and advocate the use of frameworks that allow the integration of a heterogeneous number of tools in order to collaborate and exchange data while maintaining their independence. This can help project managers who cannot afford to buy expensive integrated platforms to construct their own platform using the standalone tools at their disposal. Teams collaborating on the same task and using different tools can also continue to do so, thus capitalizing on their knowledge of the tools that they are already familiar with. The first tool is called Enabler Framework and proposed by Sinha et al. (2007). Although this tool was never released, it is to the best of our knowledge the first integrating framework to be proposed in literature. The other is PSW, which was proposed by Eskeli et al. (2011) as part of the ITEA PRISMA project. The latter has been tested by the project partners, and according to their website, they have achieved major benefits without making any major changes to their tool’s infrastructure (2011).

The limitations discussed in “Threats to validity” section show clear paths for future work on the subject. The ability to consult other digital libraries will greatly enhance the accuracy of the results. Including more tools in the study via search methods other than systematic queries in digital libraries may also be more rewarding. A more fruitful process would, for example, be to use manual research via the Internet or to carry out surveys with collaborators from industry (de Gea et al. 2011, 2012). Limited by the number and availability of the 102 identified tools, only an empirical validation of a set of tools can be conducted. A set of tools providing the most support to group interaction and covering the whole SPM process could be selected and evaluated in order to assess their usefulness for practitioners. Further investigation into the lack of tools in DM, RM and Me will be conducted to identify challenges related to GSD and a tool will be developed to counter them.

## Appendix 1

Appendix 1 provides detailed information concerning the selected articles. The information provided is the publication source and channel in Table 10. The year of publication, research type and approach, along with a detailed quality score for each selected article are provided in Table 9.

**Table 9:** Classification of selected studies, publication year and detailed quality assessment score

Article	Type	Approach	Quality assessment				
			QA1	QA2	QA3	QA4	Score
Sinha et al. (2006)	Solution proposal	Experiment	Yes	Yes	Yes	Q1	5.0
Ramasubbu and Balan (2012)	Solution proposal	Case study	Yes	Yes	Yes	CORE A*	5.0
Treude and Storey (2012)	Evaluation research	Case study	Yes	Yes	Yes	Q1	5.0
Niinimaki et al. (2010)	Evaluation research	Case study	Yes	Yes	Yes	Q2	4.5
Gorton et al. (1997b)	Solution proposal	Experiment	Yes	Yes	Yes	CORE A	4.5
Palacio et al. (2011)	Solution proposal	Experiment	Yes	Yes	Yes	Q3	4.0
Lanubile et al. (2010)	Review	Other	Yes	Yes	No	Q1	4.0
Portillo-Rodríguez et al. (2012)	Review	Other	Yes	Yes	No	Q1	4.0
Persson et al. (2009)	Solution proposal	Experiment	Yes	Yes	Yes	Q3	4.0
Bowen and Maurer (2002)	Solution proposal	Theory	Yes	Yes	No	CORE A*	4.0
Al-Ani et al. (2014)	Evaluation research	Survey	Yes	No	Yes	Q1	4.0
Samoilenko and Nahar (2012a)	Evaluation research	Theory	Yes	Yes	No	CORE A	3.5
Samoilenko and Nahar (2012b)	Evaluation research	Theory	Yes	Yes	No	CORE A	3.5
Scharff et al. (2010)	Evaluation research	Experiment	Yes	Yes	Yes	CORE C	3.5
Costa et al. (2010)	Review	Other	Yes	Yes	No	CORE A	3.5
Spanjers et al. (2006)	Evaluation research	Case study	Yes	Yes	Yes	CORE C	3.5
Goedicke et al. (2000)	Solution proposal	Theory	Yes	Yes	No	CORE A	3.5
Herring and Rees (2001)	Evaluation research	Case study	Yes	Yes	Yes	CORE C	3.5
Sakthivel (2005)	Other	Other	Yes	Yes	No	Q2	3.5
Dullemond and van Gameren (2012)	Solution proposal	Case study	Yes	Yes	Yes	CORE C	3.5
Prause et al. (2010)	Evaluation research	Survey	Yes	Yes	Yes	CORE C	3.5
Dullemond et al. (2010)	Solution proposal	Experiment	Yes	Yes	Yes	CORE C	3.5
Mullick et al. (2006)	Evaluation research	Experiment	Yes	Yes	Yes	CORE C	3.5
Niinimaki and Lassenius (2008)	Evaluation research	Case study	Yes	Yes	Yes	CORE C	3.5
Tell and Ali Babar (2011a)	Solution proposal	Theory	Yes	Yes	No	CORE A	3.5
Liukkunen et al. (2010)	Evaluation research	Case study	Yes	Yes	Yes	CORE C	3.5
Jaanu et al. (2012)	Evaluation research	Survey	Yes	Yes	Yes	CORE C	3.5
Gupta and Fernandez (2011)	Solution proposal	Survey	Yes	Yes	Yes	CORE C	3.5
Geisser et al. (2007)	Solution proposal	Experiment	Yes	Yes	Yes	CORE C	3.5
Winkler et al. (2010)	Evaluation research	Experiment	Yes	No	Yes	CORE A	3.5
Dullemond and van Gameren (2013)	Solution proposal	Survey	Yes	Yes	Yes	CORE C	3.5
Miyamoto et al. (2012)	Solution proposal	Experiment	Yes	Yes	Yes	No	3.0
Thissen et al. (2007)	Evaluation research	Case study	Yes	No	Yes	CORE B	3.0
Gorton et al. (1997a)	Solution proposal	Experiment	Yes	Yes	Yes	No	3.0
Lam and Maheshwari (2001)	Solution proposal	Theory	Yes	Yes	No	CORE B	3.0
Surjaputra and Maheshwari (1999)	Solution proposal	Theory	Yes	Yes	No	CORE B	3.0
Simmons and Ma (2006)	Solution proposal	Theory	Yes	Yes	No	CORE B	3.0
Prikladnicki et al. (2012)	Review	Other	Yes	No	No	Q1	3.0
Giuffrida and Dittrich (2011)	Evaluation research	Case study	Yes	Yes	Yes	No	3.0
Beecham et al. (2012)	Solution proposal	Experiment	Yes	Yes	Yes	No	3.0
Lanubile et al. (2013)	Evaluation research	Theory	Yes	No	No	Q1	3.0
Portillo-Rodríguez et al. (2014)	Review	Other	Partially	Yes	No	Q1	3.0
Eskeli et al. (2011)	Solution proposal	Theory	Yes	Yes	No	CORE C	2.5
Wesslin et al. (2011)	Other	Other	Partially	Yes	No	CORE A	2.5
Portillo-Rodríguez et al. (2010)	Review	Other	Yes	Yes	No	CORE C	2.5
Monasor et al. (2010b)	Solution proposal	Theory	Yes	Yes	No	CORE C	2.5
Lamersdorf and Munch (2009)	Solution proposal	Theory	Yes	Yes	No	CORE C	2.5
Martignoni (2009)	Review	Other	Yes	Yes	No	CORE C	2.5
Sinha et al. (2007)	Solution proposal	Theory	Yes	Yes	No	CORE C	2.5
Cook et al. (2005)	Solution proposal	Experiment	Yes	No	Yes	CORE C	2.5
Giuffrida and Dittrich (2013)	Review	Other	Yes	No	No	Q2	2.5
Cataldo et al. (2009)	Solution proposal	Theory	Yes	Yes	No	CORE C	2.5
Mak and Kruchten (2006)	Solution proposal	Theory	Yes	Yes	No	CORE C	2.5
Simmons (2003)	Solution proposal	Theory	Yes	Yes	No	CORE C	2.5
Clear (2009)	Solution proposal	Experiment	Yes	No	Yes	CORE C	2.5
Vathsavayi et al. (2014)	Solution proposal	Theory	Yes	Yes	No	CORE C	2.5
Costa and Murta (2013)	Review	Other	Yes	Yes	No	CORE C	2.5
Chubov and Droujkov (2007)	Other	Other	Partially	No	Yes	CORE B	2.0
Monasor et al. (2010a)	Solution proposal	Theory	Yes	Yes	No	No	2.0
de Souza and Fonseca (2007)	Solution proposal	Theory	Yes	Yes	No	No	2.0
Garrido et al. (2012)	Solution proposal	Theory	Yes	Yes	No	No	2.0
Pesola et al. (2011)	Solution proposal	Theory	Yes	Yes	No	No	2.0
Aranda et al. (2011)	Solution proposal	Theory	Yes	Yes	No	No	2.0
Wu (2012)	Evaluation research	Theory	Yes	Yes	No	No	2.0
Jiménez et al. (2009)	Review	Other	Partially	No	No	Q2	1.5
Dullemond et al. (2009)	Other	Other	Yes	No	No	CORE C	1.5
Aranda et al. (2008)	Other	Other	Yes	No	No	CORE C	1.5
da Silva et al. (2010)	Review	Other	Yes	No	No	CORE C	1.5
Aranda et al. (2006)	Other	Other	Yes	No	No	CORE C	1.5
Murdoch and Astley (2004)	Evaluation research	Theory	Partially	No	No	CORE B	1.0
Salger et al. (2010)	Other	Other	Partially	No	No	CORE C	0.5
Paulish (2006)	Other	Other	Partially	No	No	CORE C	0.5
Ali et al. (2010)	Other	Other	Partially	No	No	CORE C	0.5
van Hillegersberg and Herrera (2007)	Other	Other	Partially	No	No	No	0.0
Wang et al. (2012)	Review	Other	Partially	No	No	No	0.0
Tell and Ali Babar (2011b)	Solution proposal	Theory	Partially	No	No	No	0.0

**Table 10:** Publication channel and source

Pub. source	Pub. channel	Articles	Number
IEEE International Conference on Global Software Engineering, ICGSE	Conference	Ali et al. (2010), Aranda et al. (2006), Cataldo et al. (2009), Clear (2009), Dullemond and van Gameren (2012), Dullemond et al. (2009, 2010), Gupta and Fernandez (2011), Jaanu et al. (2012), Lamersdorf and Munch (2009), Liukkunen et al. (2010), Martignoni (2009), Mullick et al. (2006), Niinimaki and Lassenius (2008), Niinimaki et al. (2010), Paulish (2006), Portillo-Rodríguez et al. (2010), Prause et al. (2010), Salger et al. (2010), da Silva et al. (2010), Sinha et al. (2007), Spanjers et al. (2006), Dullemond and van Gameren (2013), Costa and Murta (2013)	24
International Conference on Global Software Engineering Workshops, ICGSEW	Workshop	Beecham et al. (2012), Garrido et al. (2012), Giuffrida and Dittrich (2011), Pesola et al. (2011), Tell and Ali Babar (2011b)	5
IEEE Computer Society’s IEEE Software	Journal	Prikladnicki et al. (2012), Sinha et al. (2006), Lanubile et al. (2013, 2010)	4
Information and Software Technology	Journal	Portillo-Rodríguez et al. (2012), Giuffrida and Dittrich (2013), Sakthivel (2005), Al-Ani et al. (2014)	4
International Conference on Software Engineering Advances, ICSEA	Conference	Geisser et al. (2007), Eskeli et al. (2011), Scharff et al. (2010)	3
Portland International Center for Management of Engineering and Technology Conferences, PICMET	Conference	Samoilenko and Nahar (2012a, b), Wesslin et al. (2011)	3
Collaboration Researchers International Working Group Conferences, CRIWG	Conference	Aranda et al. (2011), Monasor et al. (2010a)	2
International Conference on Evaluation and Assessment in Software Engineering, EASE	Conference	Costa et al. (2010), Winkler et al. (2010)	2
International Conference on Software Engineering, ICSE	Conference	Bowen and Maurer (2002), Ramasubbu and Balan (2012)	2
Advances in Software Engineering	Journal	Jiménez et al. (2009)	1
Asia-Pacific Software Engineering Conference, APSEC	Conference	Cook et al. (2005)	1
IEEE/ACM International Conference on Automated Software Engineering, ASE	Conference	Tell and Ali Babar (2011a)	1
BT Technology	Journal	Gorton et al. (1997a)	1
Canadian Conference on Electrical and Computer Engineering, CCECE	Conference	Mak and Kruchten (2006)	1
International Workshop on Cooperative and Human Aspects of Software Engineering, CHASE	Workshop	Wang et al. (2012)	1
IEEE Computer Software and Applications Conference, COMPSAC	Conference	Lam and Maheshwari (2001)	1
IEEE Engineering Management	Journal	Persson et al. (2009)	1
International Workshop on Future Trends of Distributed Computing Systems, FTDCS	Workshop	Simmons (2003)	1
Hawaii International Conference on System Sciences, HICSS	Conference	Gorton et al. (1997b)	1
International Conference on Cognitive Informatics, ICCI	Conference	Aranda et al. (2008)	1
IEEE International Conference on Tools with Artificial Intelligence, ICTAI	Conference	Simmons and Ma (2006)	1
World Multiconference on Systemics, Cybernetics and Informatics, ISAS-SCI	Conference	Herring and Rees (2001)	1
International Conference on Information Technology Based Higher Education and Training, ITHET	Conference	Monasor et al. (2010b)	1
ACM Special Interest Group on Management Information Systems Conference on Computer Personnel Research, SIGMIS CPR	Conference	Thissen et al. (2007)	1
IEEE International Conference on Systems, Man and Cybernetics, SMC	Conference	Murdoch and Astley (2004)	1
Software Engineering, IEEE	Journal	Treude and Storey (2012)	1
IET Software	Journal	Palacio et al. (2011)	1
IEEE International Conference on Service Operations and Logistics, and Informatics, SOLI	Conference	Wu (2012)	1
Service Research and Innovation Institute Global Conference, SRII	Conference	Miyamoto et al. (2012)	1
International Conference on Tools and Algorithms for Construction and Analysis of Systems, TACAS	Conference	Goedicke et al. (2000)	1
Conferences on Tools for Managing Globally Distributed Software Development and Requirements Management in Distributed Projects, TOMAG + REMIDI	Conference	van Hillegersberg and Herrera (2007)	1
Workshop de Desenvolvimento Distribuido de Software, WDDS	Workshop	de Souza and Fonseca (2007)	1
Workshop on Enabling Technologies on Infrastructure for Collaborative Enterprises, WETICE	Workshop	Surjaputra and Maheshwari (1999)	1
International conference on Agile processes in software engineering and extreme programming, XP	Conference	Chubov and Droujkov (2007)	1
IEEE International Conference on Computer and Information Technology, ICCIT	Conference	Vathsavayi et al. (2014)	1
Information Sciences	Journal	Portillo-Rodríguez et al. (2014)	1

## Appendix 2

Appendix 2 provides detailed information on the tools that have been listed by means of this SMS, which SPM area they support, their license, their type and which of the dimensions of the 3C collaboration model they focus on. This information is displayed in Table 12 and the acronyms used are explained in Table 11.

**Table 11:** Acronyms used in 12

Acronyms							
Project processes		License		Type		3C dimension	
PP	Project planning	Res.	Research	Plat.	Platform	Comm.	Communication
PA	Project assessment and control	Cml	Commercial	Env.	Environment	Coop.	Cooperation
DM	Decision management			Fram.	Framework	Coor.	Coordination
RM	Risk management			SAT	Stand alone tool		
CM	Configuration management						
IM	Information management						
Me	Measurement

**Table 12:** SPM tools used in GSD

Tool name	P	P	D	R	C	I	M	License	Type	Comm.	Coop.	Coor.	Paper
	P	A	M	M	M	M	e						
“Miyamoto et al.’s Tool”						x		Res.	SAT	x			Miyamoto et al. (2012)
Fonseca’s Tool		x						Res.	SAT			x	Portillo-Rodríguez et al. (2012, 2014)
Vathsavayi et al.’s Tool	x							Res.	SAT			x	Vathsavayi et al. (2014)
ABC4GSD					x	x		Res.	SAT		x	x	Tell and Ali Babar (2011b)
ActiveCollab	x	x						Cml	Plat.	x	x	x	Lanubile et al. (2010), Portillo-Rodríguez et al. (2010, 2012), Prikladnicki et al. (2012)
ADAMS					x			Res.	SAT		x		Portillo-Rodríguez et al. (2012), Costa and Murta (2013)
Adobe ConnectNow						x		Cml	SAT	x	x		Portillo-Rodríguez et al. (2012)
Ariadne		x						Res.	Plugin		x	x	Portillo-Rodríguez et al. (2012, 2014), Al-Ani et al. (2014)
Assembla	x							Cml	Env.		x	x	Portillo-Rodríguez et al. (2010), Portillo-Rodríguez et al. (2012)
Atlassian Bitbucket					x			Cml	SAT		x		Martignoni (2009)
Atlassian Jira		x						Cml	SAT	x	x	x	Lanubile et al. (2010), Prikladnicki et al. (2012), Portillo-Rodríguez et al. (2010), Martignoni (2009)
Augur		x			x			Res.	SAT			x	Portillo-Rodríguez et al. (2012)
BaseCamp	x	x				x		Cml	SAT	x	x	x	Prikladnicki et al. (2012)
Bazaar					x			Free	SAT		x		Prikladnicki et al. (2012)
BSCW						x		Free	SAT	x	x	x	Portillo-Rodríguez et al. (2012)
BugZilla		x						Free	SAT		x	x	Lanubile et al. (2010), Prikladnicki et al. (2012), Martignoni (2009)
CASI					x			Res.	Plugin			x	Portillo-Rodríguez et al. (2012), Costa and Murta (2013)
CAWS						x		Res.	SAT		x		Portillo-Rodríguez et al. (2012)
CodeBeamer	x							Cml	Plat.		x	x	Portillo-Rodríguez et al. (2012)
CodeSaw		x						Res.	SAT		x	x	Portillo-Rodríguez et al. (2014)
CVE						x		Res.	Env.	x			Portillo-Rodríguez et al. (2012)
Communico						x		Res.	SAT	x			Dullemond and van Gameren (2012), Dullemond et al. (2010)
Consensus@nywhere						x		Res.	SAT	x			Portillo-Rodríguez et al. (2012)
CruiseControl	x							Free	SAT		x	x	Portillo-Rodríguez et al. (2012)
CVS					x			Free	SAT		x		Costa et al. (2010), Prikladnicki et al. (2012), Martignoni (2009)
CWS-IM						x		Res.	SAT	x		x	Palacio et al. (2011)
Darcs					x			Free	SAT		x		Lanubile et al. (2010), Portillo-Rodríguez et al. (2012), Costa and Murta (2013)
Decision Support System			x					Res.	SAT	x			Beecham et al. (2012)
DOCTOR					x	x		Cml	SAT	x	x		Portillo-Rodríguez et al. (2012)
DPMS				x				Res.	SAT	x	x	x	Persson et al. (2009)
DPMTool			x					Res.	SAT	x			Garrido et al. (2012)
Dr.Project		x			x	x		Free	SAT	x	x		Portillo-Rodríguez et al. (2012)
DSPMTool		x			x	x		Res.	Plat.	x			Lam and Maheshwari (2001)
Eclipse Help System						x		Free	SAT	x			Lanubile et al. (2010)
eConference						x		Res.	SAT	x			Lanubile et al. (2010), Portillo-Rodríguez et al. (2012)
Enabler Framework	x	x		x		x		Res.	Fram.		x	x	Sinha et al. (2007)
FASTBullet		x						Res.	SAT			x	Wang et al. (2012), Portillo-Rodríguez et al. (2012)
FriendFeed						x		Free	SAT	x			Portillo-Rodríguez et al. (2012), Costa and Murta (2013)
GENESIS		x				x		Free	SAT	x		x	Monasor et al. (2010a, b)
Git					x			Free	SAT		x		Lanubile et al. (2010), Prikladnicki et al. (2012), Portillo-Rodríguez et al. (2012), Costa and Murta (2013)
Google Docs						x		Free	SAT		x		Portillo-Rodríguez et al. (2012)
Google Groups								Free	SAT	x			Portillo-Rodríguez et al. (2012)
GWSE	x	x				x		Res.	Env.		x		Gorton et al. (1997a, b)
iBistro					x	x		Res.	SAT	x	x	x	Monasor et al. (2010a, b), Portillo-Rodríguez et al. (2012)
IBM Jazz Source Control					x			Cml	SAT		x		Costa and Murta (2013)
IBM Lotus Notes						x		Cml	Plat.	x	x		Lanubile et al. (2010), Portillo-Rodríguez et al. (2010)
IBM Rational ClearCase		x						Cml	SAT		x		Martignoni (2009), Portillo-Rodríguez et al. (2012), Costa and Murta (2013)
IBM Rational Team Concert	x				x	x		Cml	Fram.	x		x	Scharff et al. (2010), Portillo-Rodríguez et al. (2010)
IRIS		x						Res.	Env.		x	x	Dullemond and van Gameren (2013)
IssuePlayer	x	x						Res.	SAT			x	Portillo-Rodríguez et al. (2012)
Knowfact						x		Res.	SAT		x		Portillo-Rodríguez et al. (2012)
Knowledge Tree						x		Cml	SAT	x	x		Lanubile et al. (2010)
LiveNet						x		Free	SAT	x	x	x	Portillo-Rodríguez et al. (2012)
Lotus Quickr					x	x		Cml	SAT	x	x	x	Portillo-Rodríguez et al. (2012)
Lotus Sametime						x		Cml	SAT	x	x		Portillo-Rodríguez et al. (2012), Niinimaki and Lassenius (2008)
Mantis		x						Free	SAT		x	x	Portillo-Rodríguez et al. (2012)
MasePlanner	x							Res.	SAT		x	x	Portillo-Rodríguez et al. (2012)
Mercurial					x			Free	SAT		x		Lanubile et al. (2010), Portillo-Rodríguez et al. (2012), Costa and Murta (2013)
Microsoft Office Communicator						x		Cml	SAT	x			Portillo-Rodríguez et al. (2012), Niinimaki and Lassenius (2008)
Microsoft Office LiveMeeting						x		Cml	SAT	x			Al-Ani et al. (2014)
Microsoft Project	x							Cml	SAT		x	x	Prikladnicki et al. (2012)
Microsoft Sharepoint						x		Cml	SAT	x	x		Lanubile et al. (2010), Martignoni (2009), Portillo-Rodríguez et al. (2012)
MILOS ASE	x	x			x	x	x	Res.	Env.		x	x	Bowen and Maurer (2002), Portillo-Rodríguez et al. (2012)
MiraMar 2.0						x		Res.	Env.	x	x		Portillo-Rodríguez et al. (2012)
Moin Moin						x		Free	SAT	x			Portillo-Rodríguez et al. (2012)
MPK 2.0						x		Free	Env.	x	x		Portillo-Rodríguez et al. (2012)
MUDABlue					x			Res.	SAT		x		Portillo-Rodríguez et al. (2012)
MULTIMIND						x		Res.	SAT		x		Portillo-Rodríguez et al. (2012)
NextMove	x				x	x		Res.	SAT	x	x	x	Mak and Kruchten (2006), Costa et al. (2010), da Silva et al. (2010)
P2P Conference						x		Res.	SAT	x			Portillo-Rodríguez et al. (2012)
Palantir					x			Res.	Plugin		x	x	Wang et al. (2012), Portillo-Rodríguez et al. (2012), Costa and Murta (2013)
PAMPA2		x		x			x	Res.	SAT		x		Simmons (2003)
Perforce					x			Cml	SAT		x		Portillo-Rodríguez et al. (2012), Costa and Murta (2013)
PSW	x	x			x	x		Res.	Fram.	x	x	x	Eskeli et al. (2011), Pesola et al. (2011)
RepoGuard					x			Res.	Fram.		x		Portillo-Rodríguez et al. (2012)
Saperion ECM						x		Cml	Plat.		x		Martignoni (2009), Portillo-Rodríguez et al. (2012)
SCARAB		x						Free	SAT		x	x	Niinimaki and Lassenius (2008)
Skype						x		Cml	SAT	x			Martignoni (2009), Al-Ani et al. (2014)
SEES		x			x		x	Res.	SAT			x	Simmons and Ma (2006)
Sparx Systems Enterprise Architect					x			Cml	Plat.		x	x	Salger et al. (2010)
Subclipse					x			Res.	Plugin		x		Lanubile et al. (2010), Portillo-Rodríguez et al. (2010)
SubVersion					x			Free	SAT		x		Lanubile et al. (2010), Prikladnicki et al. (2012), Portillo-Rodríguez et al. (2010, 2012), Martignoni (2009), Costa and Murta (2013)
Sysiphus					x			Res.	Env.		x		Costa and Murta (2013)
TAMRI	x							Res.	SAT			x	Lamersdorf and Munch (2009), Costa et al. (2010), Portillo-Rodríguez et al. (2012), da Silva et al. (2010)
TeamSpace						x		Res.	SAT	x			Costa et al. (2010), da Silva et al. (2010)
Tesseract						x		Res.	SAT	x			Wang et al. (2012), Portillo-Rodríguez et al. (2012, 2014)
Theseus	x					x		Res.	SAT		x		de Souza and Fonseca (2007)
Tortoises SVN					x			Free	SAT		x		Portillo-Rodríguez et al. (2012)
Trac		x						Free	SAT		x	x	Prikladnicki et al. (2012), Portillo-Rodríguez et al. (2010)
TraVis		x						Res.	SAT		x	x	Geisser et al. (2007), Portillo-Rodríguez et al. (2014)
Trusty						x		Res.	SAT	x		x	Aranda et al. (2011)
Twiki						x		Free	Plat.	x	x		Portillo-Rodríguez et al. (2010), Chubov and Droujkov (2007)
Twitter						x		Free	SAT	x			Portillo-Rodríguez et al. (2012)
Virtual Safe					x			Cml	SAT		x		Martignoni (2009)
WebEx						x		Cml	SAT	x			Lanubile et al. (2010), Portillo-Rodríguez et al. (2012)
WikiDev2.0					x			Res.	SAT	x	x		Portillo-Rodríguez et al. (2012)
Workspace 3D						x		Cml	SAT	x	x		Lanubile et al. (2010)
Workspace Activity Viewer	x	x						Res.	SAT		x		Lanubile et al. (2010), Portillo-Rodríguez et al. (2012)
WorldView		x						Res.	SAT		x	x	Lanubile et al. (2010), Portillo-Rodríguez et al. (2012, 2014), Prikladnicki et al. (2012)
Xerox Docushare						x		Cml	SAT		x		Martignoni (2009), Portillo-Rodríguez et al. (2012)
Xplanner	x							Free	SAT		x	x	Portillo-Rodríguez et al. (2012)
Yahoo Messenger						x		Cml	SAT	x			Portillo-Rodríguez et al. (2012)

## Appendix 3

### Command search queries used for IEEE Xplore

- “Document Title”:((global OR distributed OR outsourcing OR located OR offshore OR collaborative) AND (software) AND (development OR engineering OR improvement OR project) OR GSD OR DSD OR GSE OR CSE) AND (tool OR technology)
- “Abstract”:((global OR distributed OR outsourcing OR located OR offshore OR collaborative) AND (software) AND (development OR engineering OR improvement OR project) OR GSD OR DSD OR GSE OR CSE) AND (tool OR technology)
- “Author Keywords”:((global OR distributed OR outsourcing OR located OR offshore OR collaborative) AND (software) AND (development OR engineering OR improvement OR project) OR GSD OR DSD OR GSE OR CSE) AND (tool OR technology)

### Command search queries used for ScienceDirect

- tak ((global OR distributed OR outsourcing OR located OR offshore OR collaborative) AND (software) AND (development OR engineering OR improvement OR project) OR GSD OR DSD OR GSE OR CSE) AND (tool OR technology)

### Command search queries used for ACM

- (Title:((global OR distributed OR outsourcing OR located OR offshore OR collaborative) AND (software) AND (development OR engineering OR improvement OR project) OR GSD OR DSD OR GSE OR CSE) AND (tool OR technology))
- (Abstract:((global OR distributed OR outsourcing OR located OR offshore OR collaborative) AND (software) AND (development OR engineering OR improvement OR project) OR GSD OR DSD OR GSE OR CSE) AND (tool OR technology))
- (Keywords:((global OR distributed OR outsourcing OR located OR offshore OR collaborative) AND (software) AND (development OR engineering OR improvement OR project) OR GSD OR DSD OR GSE OR CSE) AND (tool OR technology))

### Command search queries used for GoogleScholar

- allintitle:((global OR distributed OR outsourcing OR located OR offshore OR collaborative) AND (software) AND (development OR engineering OR improvement OR project) OR GSD OR DSD OR GSE OR CSE) AND (tool OR technology)

### Command search queries used for DBLP

Research was carried out by typing all possible combinations of the specified search query as DBLP search interface does not offer the possibility to use command search query.
